# Testing strategies for couple engagement in prevention of mother-to-child transmission of HIV and family health in Kenya: study protocol for a randomized controlled trial

**DOI:** 10.1186/s13063-020-04956-1

**Published:** 2021-01-06

**Authors:** Zachary Kwena, Liza Kimbo, Lynae A. Darbes, Abigail M. Hatcher, Anna Helova, George Owino, Harsha Thirumurthy, Elizabeth A. Bukusi, Thomas Braun, Meredith Kilgore, Maria Pisu, Ashutosh Tamhane, Van T. Nghiem, Kawango Agot, Torsten B. Neilands, Janet M. Turan

**Affiliations:** 1 Research, Care and Treatment Programme, Centre for Microbiology Research, Kenya Medical Research Institute, Nairobi, Kenya; 2 Department of Health Care Organization and Policy, School of Public Health, University of Alabama at Birmingham, Birmingham, AL USA; 3 Center for Sexuality and Health Disparities, Department of Health Behavior and Biological Sciences, School of Nursing, University of Michigan, Ann Arbor, MI USA; 4 School of Public Health, University of Witwatersrand, Johannesburg, South Africa; 5 Department of Medical Ethics & Health Policy, Perelman School of Medicine, University of Pennsylvania, Philadelphia, PA USA; 6 Department of Biostatistics, School of Public Health, University of Michigan, Ann Arbor, MI USA; 7 Division of Preventive Medicine, Department of Medicine, School of Medicine, University of Alabama at Birmingham, Birmingham, AL USA; 8 Department of Medicine, School of Medicine, University of Alabama at Birmingham, Birmingham, AL USA; 9 Impact Research and Development Organization, Kisumu, Kenya; 10 Division of Prevention Sciences, Department of Medicine, University of California San Francisco, San Francisco, USA

**Keywords:** Antiretroviral therapy (ART), Prevention of mother-to-child transmission (PMTCT), Couple relationships, Couple home testing and counseling (CHTC), HIV self-testing kits (HIVST), Intimate partner violence (IPV), Randomized controlled trial (RCT)

## Abstract

**Background:**

HIV-related maternal deaths and HIV infection among infants remain unacceptably high across sub-Saharan Africa despite increased antenatal care attendance and provision of antiretroviral therapy to pregnant women. In the Jamii Bora (“Better Family” in Swahili) Study, we seek to test the efficacy of an interdependence theory-based couple intervention. The intervention reaches pregnant women and male partners through home visits by male-female pairs of lay health workers. The aim is to increase access to home-based couples’ HIV testing and counseling services to improve family health.

**Methods:**

This is a three-arm randomized control trial among 1080 pregnant women 15 years of age or older, living with their male partners, and who have not undergone couples’ HIV testing and counseling in Kisumu and Migori Counties in Kenya. Couples will be randomized into three groups: home-based couple visits, HIV self-testing kits for couple use, or standard care (male partner clinic invitation letters). Participants will be followed up to 18 months postpartum. The study has three aims: in aim 1, we will determine the effects of the intervention on our primary outcome of couple HIV testing, compared to HIV self-testing kits and standard care; in aim 2, we will examine the intervention impact on HIV prevention behaviors, facility delivery, and postnatal healthcare utilization, as well as secondary health outcomes of maternal viral suppression and HIV-free child survival up to 18 months for couples living with HIV; and in aim 3, we will compare the cost-effectiveness of the home-based couple intervention to the less resource-intensive strategies used in the other two study arms. Assessments with couples are conducted at baseline, late pregnancy, and at months 3, 6, 12, and 18 after birth.

**Discussion:**

The results from this study will inform decision-makers about the cost-effective strategies to engage pregnant couples in the prevention of mother-to-child transmission and family health, with important downstream benefits for maternal, paternal, and infant health.

**Trial registration:**

ClinicalTrials.gov NCT03547739. Registered on May 9, 2018

**Supplementary Information:**

The online version contains supplementary material available at 10.1186/s13063-020-04956-1.

## Administrative information

**Table:** 

Title	Testing Strategies for Couple Engagement in PMTCT and Family Health in Kenya: study protocol for a randomized controlled trial
Trial Registration	ClinicalTrials.gov NCT03547739
Protocol Version	Jamii Bora R01; Protocol Amendment 4, version 6.0 April 04, 2020.
Funding	U.S. NIH/NIMH grant R01MH116736
Author details	Research, Care and Treatment Programme, Centre for Microbiology Research, Kenya Medical Research Institute, Nairobi, Kenya Department of Health Care Organization and Policy, School of Public Health, University of Alabama at Birmingham, Birmingham, AL, USA Center for Sexuality and Health Disparities, and Department of Health Behavior and Biological Sciences, School of Nursing, University of Michigan, Ann Arbor, MI, USA School of Public Health, University of Witwatersrand, Johannesburg, South Africa Department of Medical Ethics & Health Policy, Perelman School of Medicine, University of Pennsylvania, Philadelphia, PA, USA Department of Biostatistics, School of Public Health, University of Michigan, Ann Arbor, MI, USA Division of Preventive Medicine, Department of Medicine, School of Medicine University of Alabama at Birmingham, Birmingham, AL, USA Department of Medicine, School of Medicine University of Alabama at Birmingham, Birmingham, AL, USA Impact Research and Development Organization, Kisumu, Kenya Division of Prevention Sciences, Department of Medicine, University of California San Francisco, CA, USA
Name and contact information for the trial sponsor	Janet M. Turan, PhD, MPHProfessor, Department of Health Care Organization and Policy School of Public Health, University of Alabama at Birmingham Ryals PH Building, 517D email: jmturan@uab.edu, jmturan@gmail.com office: (205) 934-6780, cell: (205) 639-3302
Role of sponsor	The Principal Investigators (Turan and Darbes) are responsible for study design, oversight of collection, management, analysis and interpretation of data; review of the report and decision on submission of the report for publication; and hold ultimate authority over these activities. The content is solely the responsibility of the authors and does not necessarily represent the official views of the funder, the NIH/National Institute of Mental Health. The funders had no role in study design, data collection, and analysis, decision to publish, or preparation of the manuscript.

## Introduction

### Background and rationale

The provision of antiretroviral therapy (ART) improves maternal health and is a key pillar for the elimination of mother-to-child transmission of HIV [1]. However, HIV-related maternal deaths and HIV infection among infants remain unacceptably high across sub-Saharan Africa [2]. This is particularly true in Kenya, where antenatal care attendance is high [3], but crucial drop-offs occur in the uptake of and adherence to key maternal and child health and prevention of mother-to-child transmission (PMTCT) services [4]. In 2017, 57% of women presenting at antenatal clinics were tested for HIV with 76% of pregnant HIV-positive women receiving ART for PMTCT [3]. Even though HIV testing rates have been increasing over time, 12% of pregnant women in Kenya transmitted HIV to their infants in 2017 [4]. Among those who initially access PMTCT, rates of subsequent dropout are high, reaching rates of 42% after 12 months in some parts of sub-Saharan Africa [5].

Throughout sub-Saharan Africa, many pregnant women avoid couple HIV testing or do not adhere to PMTCT services because they fear the negative consequences of HIV for their relationship with their male partner [6, 7]. Fears and experiences of HIV-related stigma, discrimination, and violence are the common themes in narratives of pregnant women affected by HIV [8, 9] and represent key barriers to the completion of the PMTCT cascade. Women newly diagnosed with HIV in their pregnancy have been found to be less likely to remain in care [10]. It has been noted that improving the PMTCT coverage could prevent as many infant HIV deaths as would developing more effective drug regimens [9].

However, multiple social factors that contribute to dropout rates for the PMTCT cascade must be addressed to increase retention rates [8, 9]. Lack of disclosure of HIV testing and test results to male partners is a significant barrier to health service utilization by pregnant and postpartum women. In addition, non-disclosure of HIV status between partners—often resulting from fears of stigma, discrimination, and violence—has been found to limit PMTCT uptake in sub-Saharan Africa [11, 12]. Disclosure of HIV status has important benefits including gaining access to social support, lowering the risk of HIV transmission to partners, obtaining appropriate medical treatment, decreasing stress, and creating closer relationships with others [12]. Partner non-disclosure is associated with poor PMTCT adherence [13] because this lack of disclosure limits women’s ability to link and adhere to HIV care for their health. Non-disclosure also poses a risk for sexual transmission of HIV if the male partner is still HIV-negative [14], increases the odds of non-optimal adherence to PMTCT interventions [15], and heightens the risk of vertical transmission of HIV [16].

Promoting couple testing among HIV-negative pregnant women and their male partners is also essential to reducing HIV acquisition risk. As HIV transmission risk increases by more than 2-fold during pregnancy, it is likely that uninfected women and their male partners are at heightened risk of incident infection and contribute a significant number of vertically transmitted HIV cases [17], making them a crucial group for interventions [18].

Male partners are clearly a key factor in the retention of women and infants in the PMTCT continuum of care, and most PMTCT programs have not been successful in engaging men. When male partners are uninvolved in HIV testing and antenatal care, women are less likely to (1) accept ART [19], (2) deliver in a health facility, and (3) adhere to care [20]. Thus, it is unsurprising that scholars globally have advocated for engaging men in PMTCT [19, 21]. Men’s lack of involvement in HIV testing and antenatal care is compounded by gender norms that limit men’s ability to involve themselves in ANC and label ANC clinics and health facilities as “female spaces” [22]. Yet, men themselves desire more involvement in PMTCT and antenatal services [23, 24], and innovative approaches that do not involve visiting health facilities are necessary to ensure that male partner involvement occurs in a safe and supportive way [25, 26].

Couples’ HIV testing and counseling (CHTC), an evidence-based intervention, offers the potential to engage men and women but has been underutilized in the PMTCT context. Based on the evidence of the need to include both pregnant women and their male partners in PMTCT, programs across Africa have increasingly called for CHTC [15, 25]. Yet, most CHTC programs are implemented in clinics, making it unlikely that pregnant women and male partners will utilize them given poor male attendance in many settings [27]. In Kenya, while the majority of pregnant women received HIV testing, only 27% of their male partners underwent HIV testing within the last 12 months [28]. Even if women receive individual testing in antenatal care and may already know their HIV status, participating in CHTC with their partners offers a safe environment for serostatus disclosure, combined with tailored counseling and solution-building for the couple [23].

There is also a need to compare different approaches for increasing male engagement and couple testing, including innovative approaches such as HIV self-testing kits (HIVST), which is recommended for scale-up by the World Health Organization (WHO) [29]. Existing research shows a high level of acceptability and demand for HIVST across a wide range of populations and settings, as well as the accuracy of testing in the hands of lay users [30, 31]. With its increased convenience and privacy, HIVST can make it easier for pregnant women and their partners to test together. Early evidence suggests this approach is feasible, safe, and promising [32], but further research is needed to establish how health outcomes and behaviors compare to standard care and home-based couple testing interventions.

### Objectives

We propose to test the efficacy and cost-effectiveness of Jamii Bora, a home-based couple-focused intervention, to increase CHTC and disclosure and improve uptake of PMTCT and family health services. Specifically, we aim to:

Aim 1: Determine the impact of a couple-focused home-based intervention on our primary outcome of couple HIV testing for pregnant women and their male partners as compared to couple testing under HIVST and standard care

Aim 2: Examine the impact of the intervention on HIV prevention behaviors, pre-exposure prophylaxis (PrEP) and condom use, facility delivery, and postnatal healthcare utilization, as well as secondary health outcomes of maternal VL suppression and HIV-free child survival up to 18 months for couples living with HIV, as compared to providing HIV self-tests for couples and standard care.

Aim 3: Compare the cost-effectiveness of the home-based couple intervention to less resource-intensive strategies of standard care and HIV self-testing kits for couples.

### Trial design

**Fig. 1:**
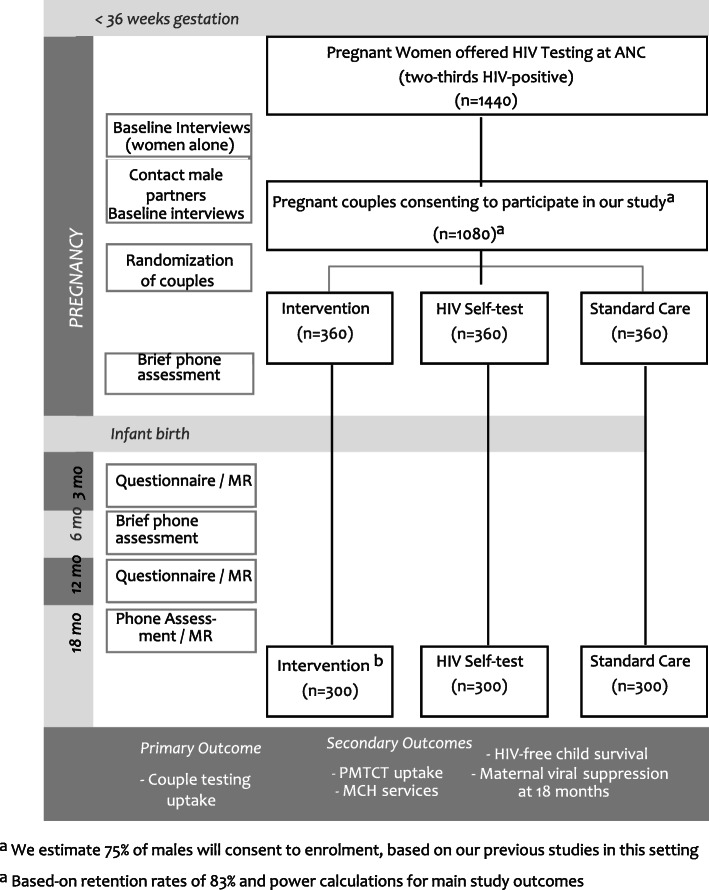
Study design

This is a three-arm couple-randomized controlled trial enrolling 1080 pregnant women attending antenatal clinics (720 HIV-positive and 360 HIV-negative at baseline) and their male partners to assess the impact and cost-effectiveness of the intervention on health behaviors and health outcomes, following the couples for up to 18 months postpartum. Figure 1 shows the schedule of couples’ assessments over the 18-month period.

## Methods: participants, interventions, and outcomes

### Study setting

This study will be conducted in 18 ANC health facilities, half in Kisumu County and the other half in Migori County. Kisumu County (19.3%) and Migori County (14.7%) are among the top four highest HIV-burden counties for adults in Kenya [33]. Maternal mortality in the counties is high with Kisumu County at 465 [34] and Migori County at 673 per 100,000 live births [35]. The two counties account for 13% of new infant HIV infections in the country and 15.13% of the need for PMTCT services [4]. Although the vast majority of women who present at ANC clinics in Kisumu and Migori counties are tested for HIV, the number of pregnant women testing for HIV at the national level has been reducing in recent years to stand at 56.8% in 2017 with a high rate of MTCT at 11.5% [3]. The two study counties report lower than national rates of MTCT at 8.7% for Kisumu and 7.2% for Migori County, and there is a significant dropout of women and infants along the PMTCT cascade [36]. The clinics in this setting provide integrated antenatal care/maternal and child health (ANC/MCH) and HIV services and are implementing the Option B+ strategy, in which all pregnant and breastfeeding women are immediately initiated on life-long ART, regardless of CD4 count or HIV disease stage.

**Fig. 2:**
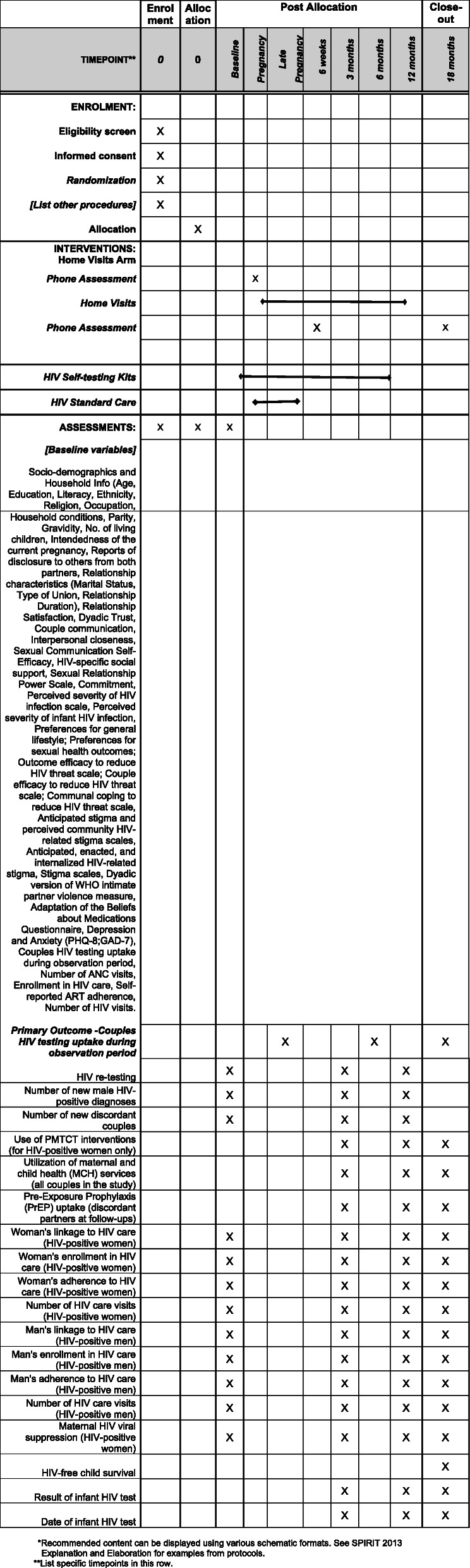
Detailed study design

We adapted the Interdependence Model of Health Behavior Change to understand the mechanisms through which this intervention may impact health outcomes (Fig. 2 [37]. This model extends beyond an individually based understanding of health behavior change (e.g., health beliefs, self-efficacy) by positing that both partners influence one another’s health decisions and behaviors and emphasizing that positive relationship dynamics, e.g., communication and commitment, are inherent to a couple’s ability to engage in joint decision-making aimed to improve health outcomes for the couple [38].

### Eligibility criteria

The following are the inclusion criteria:

- Women at 36 weeks of pregnancy or less.
- 15 years of age or older.
- Has been offered HIV testing at ANC.
- Is currently in a stable relationship with a male partner and living with that male partner.
- Has not yet participated in a couple HIV testing during this pregnancy.
- The male partner is the person identified by the pregnant woman as her primary male partner and should also be 15 years of age or older.
- Not in an HIV-positive concordant relationship.

The following are the exclusion criteria:

- Greater than 36 weeks of pregnancy
- Less than 15 years of age
- Not currently in a stable relationship with a male partner
- Does not currently live with a male partner
- Has not been offered HIV testing at ANC

Women who report recent severe intimate partner violence as assessed by the baseline questionnaire responses to the dyadic version of the WHO intimate partner violence measure [39] are included in the study assessments, but their male partners are not contacted for enrollment, and the couple is not included in the randomized part of the study.

### Informed consent

Pregnant women presenting at participating ANC clinics in Kisumu and Migori Counties who meet study the inclusion criteria are asked if they would like to participate in a study about approaches for supporting pregnant couples on family health issues (including HIV) during pregnancy and postpartum. If interested, a lay health worker obtains informed consent for study participation, conducts the baseline questionnaire, and obtains permission for contacting her male partner. With the woman’s permission, we subsequently contact her primary male partner, arrange to meet with him in a community location, obtain informed consent, and conduct his baseline questionnaire.

### Interventions/participant timeline

#### Home-based couple counseling intervention

The home-based couple intervention includes five home visits (3 main and 2 booster visits) laid out as follows: two during pregnancy, one at 6 weeks after the birth; two booster sessions, one at 6 months after the birth and one at 12 months after the birth. At each home visit, pairs of lay health workers (one male and one female) meet with the woman and her partner together at their home (or another preferred community site) and deliver key intervention elements as shown in Table 1. The key elements include discussing family health promotion, HIV-related services including offering CHTC, couple communication skills such as active listening, and active linkage to nearby clinic-based services for family health and HIV prevention and treatment services (including PrEP for discordant couples).

**Table 1:** Intervention content at each couple home visit

**Visit #**	**Visit 1**	**Visit 2**	**Visit 3**	**Visit 4**	**Visit 5**
**Timing**	Pregnancy	Later pregnancy	6 weeks postpartum	6 months postpartum	12 months postpartum
**Main family health topics**	• ANC visits • Nutrition • Malaria • Male partner support during pregnancy • IPV • Mental health	• Birth plan for HF delivery • Danger signs • Infant feeding • Male partner support for birth • What to expect	• Infant health visits and immunizations • Family planning • Male partner support after birth • Postpartum checkups	• Infant health • Infant feeding • Family planning • Male health • IPV • Mental health	• Infant feeding • Infant visits and immunizations • Family planning
**HIV-related content**	• Couple HIV testing • PMTCT^b^ • PrEP^a^ • Linkage to care^b^	• Repeat testing • PMTCT^b^ • PrEP^a^ • Linkage to care^b^	• Infant HIV testing^b^ • PMTCT^b^ • PrEP^a^ • Linkage to care^b^	• Repeat testing • PMTCT^b^ • PrEP^a^ • Linkage to care^b^	• Infant HIV testing^b^ • PMTCT^b^ • PrEP^a^ • Linkage to care^b^
**Couple relationship**	• Intro to couple relationship skills	• Use of “I” language	• Listening skills	• Negotiation skills	• Revisiting and practicing skills
**Services and linkages offered**	• Introduction of CHTC • Linkage to HIV care/PrEP	• Offer of CHTC^c^ • Linkage to HIV care/PrEP	• Offer of CHTC^c^ • Linkage to HIV care/PrEP	• Offer of CHTC^c^ • Linkage to HIV care/PrEP	• Offer of CHTC^c^ • Linkage to HIV care/PrEP

^a^For discordant couples

^b^For couples living with HIV

^c^For those who have not done CHTC yet or who need to repeat testing

Over 87% of pregnant women in this region live with their male partner [40], making home visits an optimal approach to reach the couples. Recognizing that some pregnant women may live in extended family households where privacy is difficult to maintain [41], we also identify a location in each community for couple sessions that participants may choose if privacy cannot be assured in the home. The use of lay health workers as couples’ counselors contributes to sustainability, given the inadequate availability of professional counselors in this setting.

#### Justification for the choice of comparison groups

The comparison groups are the provision of HIV self-testing kits (HIVST) for the couple (pregnant woman and male partner) and standard of care. We chose HIVST in order to compare our home-based couple intervention with a promising, but less resource-intensive, approach for encouraging couple and male partner testing. Standard of care was chosen as a comparison group in order to contrast our intervention with the approach currently being used at Kenyan ANC clinics.

#### Comparison arms

We have two comparison arms: HIV self-testing kits and standard of care. In the HIV self-testing kits arm, pregnant women receive pairs of HIVST for themselves and their male partners. Trained study staff will provide four oral fluid-based rapid HIV test kits (OraQuickRapid HIV-1/2 Antibody Test, OraSure Technologies) during pregnancy and up to four additional kits after birth. Each test is accompanied with an instruction sheet that describes the step-by-step self-testing procedures in multiple languages. The study staff provide participants with a brief demonstration of how to use the tests and encourage them to offer test kits to their partners to undertake the testing together if they feel comfortable doing so. They are also counseled on how to talk to their partners about HIV testing and the possibility of any adverse partner reactions. Women are free not to accept the test kits or to not introduce them to the male partner if they do not feel comfortable doing so. Following Kenya’s HIV testing services guidelines, participants are instructed to seek clinic-based confirmatory testing if a reactive self-test result is obtained, and an invitation for confirmatory testing at a clinic is included with each test. Participants are also encouraged to report the results of the couples’ HIVST use to the study staff.

All women in the standard care group are encouraged by the clinic staff to come to the clinic with their male partners and are routinely asked to undergo CHTC. The clinic staff use letters and other methods to invite the male partner to ANC at the next visit. Couples coming together to ANC are given priority to receive services so that they can avoid queues and rapidly be seen by clinic staff. Our initial assessments indicated that less than 25% of ANC clients participate in CHTC during ANC visits.

### Outcomes

The primary outcome measure is change in couple HIV testing uptake from baseline to 12 months postpartum. This is assessed in the questionnaires for all couples regardless of HIV status at baseline and each follow-up until 12 months postpartum and confirmed through home visitors’ reports and medical records. The change in couples’ HIV testing uptake during the observation period (from baseline to 12 months postpartum) is coded as Y/N assessed through direct observation in the intervention arm and surveys in the comparison arms. Secondary outcomes include HIV-free child survival at 18 months and maternal viral load suppression at 18 months assessed from medical records (for HIV-positive women only); the number of new male HIV-positive diagnoses coded as Y/N assessed during the observation period and confirmed from medical records; family health service utilization; men and women’s linkage to HIV care, enrollment, and adherence to care assessed from medical records; and PrEP uptake for discordant partners assessed in follow-up questionnaires and medical records.

### Sample size and power estimations

Our target populations are pregnant women identified in the ANC facilities and their male partners. We include women at 36 weeks of pregnancy or less, to have time to provide at least one home visit during pregnancy. The couple testing uptake outcome (primary outcome) will be assessed including both HIV-positive and HIV-negative women/couples. All three arms will be compared with each other (3 comparisons), so our type I error rate is 0.05/3 = 0.017 (two-sided). With three repeated measurements (baseline, 3 months, and 12 months) and compound symmetry covariance structure, the correlation between the observations on the same subject was assumed to be 0.50 as a mid-way between the range of 0.00 to 1.00. With *N* = 300 couples in each arm, our study will have > 80% power to detect statistically significant differences in couple testing uptake of 30–40% in either intervention arm and 23% in the control arm, based on CHTC rates obtained in the Jamii Bora pilot study [42] (Table 2). The secondary outcomes are HIV-free child survival at 18 months and maternal viral load suppression at 18 months. HIV-free child survival up to 18 months will be assessed only in HIV-positive women with live births, with approximately *N* = 200 in each arm. Based on prior estimates from sub-Saharan Africa [43, 44], we expect proportions ≥ 90% of HIV-free survivors in each arm. As there are two comparisons (each intervention arm vs the control arm), we set our type I error rate to 0.05/2 = 0.025 (two-sided) when calculating power. If arm 3 (standard care) has a survival rate of 91%, the study has a power of > 80% when arms 1 (home-based couple intervention) and 2 (HIVST) have survival rates of 98% or higher. The other secondary outcome of maternal viral load suppression at 18 months is assessed only in HIV-positive women with around *N* = 200 in each arm. In Table 2, if arm 3 has a maternal viral load suppression rate of 85%, the study has a power of > 80% when arms 1 and 2 have maternal viral load suppression rates of 95% or higher.

**Table 2:** Statistical power for comparison of outcomes among study arms

**1. CHTC uptake (assuming 23% uptake in the standard care arm (arm 3))**				
Proportion (%) of CHTC uptake in arm 1 or arm 2	31	33	35	37
Corresponding odds ratio (ref = arm 3)	1.50	1.65	1.80	1.97
Power (%) to detect a significant difference among arms at 0.017 significance level with an auto-correlation (rho) = **0.50**	63	84	95	99
Power (%) to detect a significant difference among arms at 0.017 significance level with an auto-correlation (rho) = **0.10**	87	97	100	100
**2. HIV-free child survival at 18 months (assuming 91% survival in the standard care arm (arm 3))**				
Proportion (%) of HIV-free survival in arm 1 or arm 2	93	95	97	99
Corresponding odds ratio (ref = arm 3)	1.31	1.88	3.20	9.79
Power (%) to detect a significant difference among arms at 0.025 significance level	7	26	62	93
**3. Maternal viral load suppression at 18 months** (**assuming viral load suppression of 85% in the standard care arm (arm 3))**				
Proportion (%) of viral load suppression in arm 1 or arm 2	90	92	94	96
Corresponding odds ratio (ref = arm 3)	1.59	2.03	2.77	4.24
Power (%) to detect a significant difference among arms at 0.025 significance level	24	49	77	94

### Recruitment

We estimated that approximately 120 HIV-positive pregnant women could be recruited from each of the 18 ANC clinics in 24 months (*n* = 960) in Kisumu and Migori Counties, Western Kenya. Using a stratified randomized design, we are recruiting HIV-negative women (*n* = 480) in balanced numbers to HIV-positive women (1:2) each month (total HIV-positive and HIV-negative *N* = 1440). Based on the rates achieved in the pilot study, we conservatively estimate a 75% participation rate for male partners in the study resulting in a total sample size of 1080 couples and estimate up to 17% loss-to-follow-up of couples by final follow-up resulting in approximately 900 couples (300 couples in each randomized group) for analysis.

### Assignment of interventions

#### Randomization

We recruit pregnant women attending ANC clinics to participate in the study until we have achieved a sample size of 1080 randomized couples—1080 women of which two thirds will be HIV-positive at baseline and 1080 male partners. Couples completing baseline interviews are randomized to one of the three approaches to increase couple engagement in HIV prevention and maternal and child health. Couples receive a sealed envelope labeled with their newly assigned study ID numbers, which contain their random assignments that are computer-generated and stratified by clinic and couple HIV status. Blocked randomization with randomly permuted block sizes is used to assure approximately equal numbers in each study arm and in each HIV status group in any given time period.

We do not randomize women or men reporting severe intimate partner violence (IPV) at baseline, indicated by responding yes to six items about severe physical or sexual violence during pregnancy as measured through the WHO multi-country study instrument [45]. Instead of randomization, these participants are referred directly to local support services and are invited to take part in the study follow-up assessments to ensure their continued safety and well-being. Participants reporting severe depression, indicated by a score of 20 or greater on the Patient Health Questionnaire (PHQ-9) [46], are randomized and referred to support services. There is limited empirical evidence that couple-based interventions could exacerbate violence or depression, but we opted to err on the conservative side (i.e., excluding high-risk women from the intervention part of the study). All participants will be asked to provide informed consent for data abstraction from their medical records.

#### Implementation

After randomization, a lay health worker obtains detailed locator information, including cellphone contacts. If randomized to the home visit intervention arm, the worker consults with them about optimal times for a home visit. The HIV self-testing kits arm consists of the distribution of pairs of self-test kits to women at up to 4 time points (twice during pregnancy and twice after the birth). The standard of care arm offers standard clinic-based services, including the standard practice of giving the pregnant woman a letter (or other modes of communication) to invite her male partner to the clinic, and the option for women and partners to return to the clinic for male partner HIV testing or CHTC.

### Data collection and management

We will collect data from multiple sources, including the following:

- ○ Baseline questionnaires on tablets will be interviewer-administered by a gender-matched interviewer on tablet computers in the participant’s preferred language (Swahili, Luo, or English).
- ○ Brief phone-based assessments of HIV testing uptake and results are administered to all participants via mobile phone including responding to a brief confidential survey on HIV testing (individual or couples) and results in late pregnancy and again at 6 months after the birth.
- ○ Follow-up questionnaires with women and male partners occur at 3 and 12 months after the birth at participants’ convenience.
- ○ A brief phone assessment at 18 months post-birth captures infant HIV status, couples’ testing behavior, and mother’s viral load if positive.
- ○ Medical records are abstracted to obtain objective data on healthcare utilization and health outcomes at late pregnancy and at 3, 12, and 18 months postpartum for all participants in the study.
- ○ A couple visit form is completed by lay health workers at each couple visit. The form will capture information on process measures such as intervention content (topics covered), participation (CHTC uptake and results), social consequences (assessments of negative life events including IPV) [47], and acceptability (satisfaction with intervention components). This form, along with records of observations of visits by supervisors, will be used to assess intervention fidelity.

Each participant is reimbursed for each assessment visit (questionnaires), but not for intervention activities, at the rate of approximately 500 Kenyan shillings (roughly 5 US dollars) paid in cash per assessment. This reimbursement is in accordance with other studies being conducted in the area. Participants in the home visit study arm receive a small gift of approximately 200 KSh value ($2 US) at each home visit, which is a cultural expectation for persons visiting the home of pregnant/postpartum couples.

Table 3 shows in detail the variables and the measurements that are assessed at various stages in the course of the study.

**Table 3:** Factors and measures to be assessed in data collection

	Factors	Study measurements	Group and timing
**Couple HIV testing (primary outcome)**	Couple HIV testing	Couple HIV testing uptake during the observation period (Y/N)*	All participants at baseline and follow-ups
**Other testing outcomes**	HIV re-testing	Re-testing for HIV during pregnancy and postpartum*	All participants at follow-ups
	New male HIV+ tests	Number of new HIV+ test results of male partners*	
	Discordant couples	Number of new serodiscordant couples identified	
**Transformation of couple motivation and couple coping**	Couple relationship dynamics	Relationship satisfaction [48], dyadic trust [49], couple communication [50], interpersonal closeness [51], Sexual Relationship Power Scale [52], commitment [53]	All participants at baseline and follow-ups
	Disclosure	Reports of disclosure to others from both partners [54]	
	HIV-related couple	Male partner support for MCH-specific social support [55], couple communal coping [56], Network of Relationships Social Provision Scale (ref)	
**Other potential moderators and mediators**	Pregnancy intendedness	One item measure of the intendedness of the current pregnancy [57]	All participants at baseline and follow-ups
	Perceptions of stigma	Anticipated stigma and perceived community HIV-related stigma scales [58, 59]	
	Stigma experience	Anticipated, enacted, and internalized HIV-related stigma [60]	HIV-positive participant
	IPV	Dyadic version of WHO intimate partner violence measure [39]	All participants at baseline and follow-ups
	HIV treatment beliefs	Adaptation of the Beliefs about Medications Questionnaire [61]	
	Depression	PHQ-8 [46]	
	Anxiety	GAD-7 [62]	
**Healthcare utilization**	PMTCT practices	Mother’s use of ARVs for PMTCT*, prophylactic ARVs for the infant*, infant feeding practices	HIV-positive women at follow-ups
	Use of MCH services	Number of ANC visits*, childbirth with a skilled attendant (Y/N), postnatal check-ups*	All participants at follow-ups
	PREP uptake	Initiation of PrEP	Discordant partners at follow-ups
	Woman’s HIV care linkage and engagement	Time to linkage in HIV care*, enrollment in HIV care (Y/N)*, self-reported ART adherence [63], number of HIV visits*	HIV-positive women at baseline and follow-ups
	Man’s HIV care linkage	Time to linkage in HIV care*, enrollment in HIV care (Y/N)*, self-reported ART adherence [63], number of HIV visits*	HIV-positive men at baseline and follow-ups
	Infant HIV testing	Date and result of infant HIV test*	Parents of HIV-exposed infants at follow-ups
**Process measures**	Intervention content	Topics covered, services delivered, referrals made during couple visits	All intervention participants at follow-ups
	Participation	Number of couple home visits completed, number of HIV self-tests received/used	
	Social consequences	Positive and negative life event measures [47]	
	Acceptability	Satisfaction with intervention components, intervention content, and mode of delivery; attitudes toward PrEP and HIV self-testing	
**Secondary health outcomes**	Viral suppression	Viral Load < 200 copies (undetectable)*	All HIV-positive participants at baseline and follow-ups
	HIV-free child survival	Child alive and HIV-free at 18 months after the birth*	

Secondary outcome measures and assessment are listed in the table above:

1. HIV re-testing [time frame: baseline, 3 months postpartum, 12 months postpartum]. Re-testing for HIV during pregnancy and postpartum during observation period assessed in the questionnaire and confirmed through medical records, completed by all participants at baseline and each follow-up until 12 months postpartum

2. Number of new male HIV-positive diagnoses [time frame: baseline, 3 months postpartum, 12 months postpartum]. Number of new HIV-positive test results of male partners during the observation period, coded as Y/N. This is assessed for all male participants at baseline and each follow-up until 12 months postpartum in the questionnaires and confirmed through medical records

3. Number of new discordant couples [time frame: baseline, 3 months postpartum, 12 months postpartum]. Number of new HIV serodiscordant couples identified during the observation period. This is assessed for all couples at baseline and each follow-up until 12 months postpartum in the questionnaires and confirmed through medical records

4. Use of PMTCT interventions (for HIV-positive women only) [time frame: 3 months postpartum, 12 months postpartum, 18 months postpartum]. Composite variable including mothers use of antiretrovirals (ARVs) (Y/N), prophylactic ARVs given to the infant (Y/N), and appropriate infant feeding practices. These are assessed in the questionnaires completed at each follow-up up to 18 months postpartum

5. Utilization of maternal and child health (MCH) services (all couples in the study) [time frame: 3 months postpartum, 12 months postpartum, 18 months postpartum]. Composite variable including having completed at least four antenatal care (ANC) visits during pregnancy (Y/N), childbirth with a skilled attendant (Y/N), and postnatal check-ups for woman (Y/N) and infant (Y/N). These are assessed in the follow-up questionnaires completed up to 18 months postpartum

6. Pre-exposure prophylaxis (PrEP) uptake (discordant partners at follow-ups) [time frame: 3 months postpartum, 12 months postpartum, 18 months postpartum]. Initiation of PrEP by discordant partners assessed at each follow-up in the questionnaires and confirmed through medical records up to 18 months after the baby’s birth

7. Woman’s linkage to HIV care (HIV-positive women) [time frame: baseline, 3 months postpartum, 12 months postpartum, 18 months postpartum]. Time to linkage to HIV care assessed for HIV-positive women at baseline and in the follow-up questionnaires completed up to 18 months postpartum and confirmed through medical records

8. Woman’s enrollment in HIV care (HIV-positive women) [time frame: baseline, 3 months postpartum, 12 months postpartum, 18 months postpartum]. Enrollment of HIV-positive women in HIV care (Y/N) assessed at baseline and in the follow-up questionnaires completed up to 18 months postpartum and confirmed through medical records

9. Woman’s adherence to HIV care (HIV-positive women) [time frame: baseline, 3 months postpartum, 12 months postpartum, 18 months postpartum]. Self-reported adherence to HIV care assessed for HIV-positive women at baseline and in the follow-up questionnaires completed up to 18 months postpartum and confirmed through medical records

10. Number of HIV care visits (HIV-positive women) [time frame: baseline, 3 months postpartum, 12 months postpartum, 18 months postpartum]. Number of HIV care visits assessed for HIV-positive women at baseline and in the follow-up questionnaires completed up to 18 months postpartum and confirmed through medical records

11. Man’s linkage to HIV care (HIV-positive men) [time frame: baseline, 3 months after baby’s birth, 12 months after baby’s birth, 18 months after baby’s birth]. Time to linkage to HIV care assessed for HIV-positive men at baseline and in the follow-up questionnaires completed up to 18 months postpartum and confirmed through medical records

12. Man’s enrollment in HIV care (HIV-positive men) [time frame: baseline, 3 months after baby’s birth, 12 months after baby’s birth, 18 months after baby’s birth]. Enrollment of HIV-positive men in HIV care (Y/N) assessed at baseline and in the follow-up questionnaires completed up to 18 months postpartum and confirmed through medical records

13. Man’s adherence to HIV care (HIV-positive men) [time frame: baseline, 3 months after baby’s birth, 12 months after baby’s birth, 18 months after baby’s birth]. Self-reported adherence to HIV care assessed for HIV-positive men at baseline and in the follow-up questionnaires completed up to 18 months postpartum and confirmed through medical records

14. Number of HIV care visits (HIV-positive men) [time frame: baseline, 3 months after baby’s birth, 12 months after baby’s birth, 18 months after baby’s birth]. Number of HIV care visits assessed for HIV-positive men at baseline and in the follow-up questionnaires completed up to 18 months postpartum and confirmed through medical records

15. Maternal HIV viral suppression (HIV-positive women) [time frame: baseline, 3 months postpartum, 12 months postpartum, 18 months postpartum]. Viral load < 200 copies (undetectable) for all HIV-positive women at baseline and 18 months postpartum through medical records

16. HIV-free child survival [time frame: 18 months after the birth]. Child alive and HIV-free at 18 months after the birth. This is assessed in a brief interview and confirmed through medical records

17. Result of infant HIV test [time frame: 3 months after birth, 12 months after birth, 18 months after birth]. Result of infant HIV test based on medical records

18. Date of infant HIV test [time frame: 3 months after birth, 12 months after birth, 18 months after birth]. Date of infant HIV test based on medical records

Data will be shared in agreement with funder (US National Institutes of Health) data sharing policies. Results will be disseminated to local stakeholders (including participants) through local presentations, regional/national/international conferences, and publications.

### Data management

Data collected on android tablets through ODK Collect (Open Data Kit 2019 Creative Commons 4.0 International License) will be aggregated for analysis using the SAS statistical software (Cary, NC, USA), version 9.4.

### Statistical methods

#### Data analyses

##### Analyses to address the primary outcome

Our primary outcome is the uptake of CHTC. We will use all longitudinal measures of couple testing in a marginal model to compare the proportions among the three study arms. We use this model because our primary interest is to estimate the population average effect of intervention participation on each outcome rather than the effect for a hypothetical average subject or couple. Moreover, within-subject and within-couple correlations among the outcomes are nuisance parameters, not quantities of interest to be modeled explicitly. Our models will include a dummy variable indicating the study group (arm 1 vs arm 3; arm 2 vs arm 3), as well as our stratifying variables and other additional covariates such as couple relationship length, if necessary. A little adjustment for confounding should be necessary due to our randomization. We will employ generalized estimating equations (GEE) with robust standard errors to obtain correct inferences because inference will be valid if the chosen correlation structure is slightly mis-specified [64]. Statistical significance will be for *p* < 0.017 for the three pairwise comparisons of the three arms to account for multiple comparisons. Between-arm differences for the other outcomes in aim 1 (mean numbers of new HIV+ diagnoses of male partners and new serodiscordant couples) will be modeled with generalized estimating equations (GEE).

##### Analyses to address secondary outcomes

Each of the secondary outcomes, including proportions of HIV prevention behaviors (PrEP and/or condom use), facility delivery, postnatal healthcare utilization, maternal VL suppression, and HIV-free infant survival up to 18 months, is binary (yes/no). Between-arm comparisons for the probabilities of these outcomes are facilitated with the same GEE models described for the primary outcome.

##### Mediation and dyadic analyses for primary and secondary outcomes

Our assessment for potential mediation and moderation will follow the approach described by Valeri and VanderWeele [65]. We will refer to the treatment effect estimates from these models as estimates A where we will then fit a second model, which takes our original GEE model and incorporates possible mediating variables, such as couple relationship dynamics and social consequences. We will refer to the parameter estimates for these three covariates as estimates B. We will then determine the direct and indirect effects of the treatment on each of the primary and secondary outcomes, with corresponding standard error estimates determined using bootstrap methods [66]. These models can also be adjusted for any potential confounders that are discovered, although we expect the randomization to account for a majority of any potential confounding.

Analyses with intact dyads enable the investigation of couple-based research questions of how relationship dynamics affect behavior change in partnerships. For example, we might investigate whether one’s own relationship satisfaction or one’s partner’s relationship satisfaction is more associated with couple testing uptake. To that end, we will extend the analyses described above to include actor and partner effects for covariates and mediators. In order to fit an actor-partner interdependence model (APIM) [67] to our data, we will change our GEE model to a random-effects model so that we can include a random effect for each couple, which will allow us to divide the variation in outcomes into within- and between-couple effects.

##### Cost-effectiveness analysis

We will assess the cost-effectiveness of the home-based couple intervention compared to two less resource-intensive strategies of HIV self-testing kits and standard of care. We hypothesize that this intervention will prove to be a cost-effective strategy compared to alternative strategies. However, cost-effectiveness might be sensitive to the intensity of services provided, levels of compensation, the extent of training, levels of adherence to ART treatment, and other attributes. We will develop a decision analysis model using the TreeAge Pro 2020 software (Williamstown, MA, USA). We will calculate the direct costs of each strategy utilizing established guidelines for costing HIV interventions [68, 69] from a program perspective using micro-costing techniques. In addition to costs, we will use data on the changes in HIV status, acquiring opportunistic infections, and mortality which are measured in disability-adjusted life year (DALY, representing a year of healthy life lost due to death or disability) to provide inputs into a Markov model. Markov models are among the most frequently used modeling techniques in clinical decision analysis and health economic evaluation and are particularly helpful when a decision analysis involves the analysis of risk over long periods of time [70, 71]. The proposed state transition models will combine Markov’s health state transitions with the probability that individuals will experience transient events that lead to either a different health state (e.g., HIV transmission) or that can carry significant costs or mortality risk, such as hospitalization for an opportunistic infection. State-transition models have been utilized in many different populations and diseases, including diabetes, cardiovascular disease, HIV, and malaria [72]. To determine the cost-effectiveness of the home-based couple intervention, we will calculate the incremental cost-effectiveness ratios (ICERs) for the intervention vs each of the comparison groups (standard care; HIV self-testing kits). The numerator of the ratio is the difference in costs expressed in US dollars (purchasing parity adjusted); the denominator is the difference in effectiveness measured in DALY. A discount rate of 3% will be applied to both costs and effectiveness. The calculated ICERs will be referenced against the WHO-recommended thresholds to determine whether the home-based couple testing intervention is very cost-effective compared to its comparators for an ICER less than gross domestic product per head, cost-effective for an ICER less than three times gross domestic product per head, or not cost-effective otherwise [73]. We will implement one-way and multiple-way sensitivity analyses to assess the robustness of the cost-effectiveness of the intervention under various uncertainties.

### Oversight and monitoring

#### DSMB

This trial is supported by a Data Safety and Monitoring Board (DSMB) independent of the funder, investigators, and competing interests. The overall responsibility of the DSMB is to protect the ethical and safety interests of participants recruited into the Jamii Bora Study while protecting as far as possible the integrity of the study and the scientific validity of the data. The DSMB will review the safety data to identify potential harm from participation in the intervention and other issues designated as necessary for their input. The DSMB will meet at 6-month intervals with the initial meeting taking place just prior to the study recruitment initiation. At all of these meetings, the DSMB will review all accrued data up to that point to assess whether the study aims are being met and to ensure that the benefits of the intervention outweigh any harm. The DSMB charter is available upon request from the trial sponsor.

#### Adverse event reporting and harms

Adverse events may include (1) extreme discomfort from HIV/blood testing, (2) relationship break-ups due to the nature of the intervention, (3) episodes of intimate partner violence, and (4) consequences of learning preliminary positive results from HIV tests. Severe adverse events include adverse pregnancy outcomes, death of a participant, and extended hospitalization. These are specified within the DSMB charter, reported to the IRBs, and discussed at regular DSMB meetings.

## Discussion

The Jamii Bora intervention aims to test the efficacy and cost-effectiveness of a home-based couple-focused intervention to facilitate CHTC and increase the use of PMTCT and family health services. The final results from this study will enhance our ability to improve HIV prevention behaviors, identification of pregnant women and male partners infected with HIV, and HIV treatment engagement and reduce the viral load for pregnant women and their male partners. These outcomes, in turn, will reduce the likelihood of vertical and horizontal HIV transmission among heterosexual couples in Western Kenya, a population at high risk for HIV. Most HIV infections in sub-Saharan Africa are occurring within primary partnerships. Thus, intervening with couples also increases the participation of men in HIV prevention activities, which have previously focused on women (especially in the antenatal context), and could contribute to a shift in community norms regarding gender and couple relations. The ability to reduce the likelihood of HIV transmission within partnerships, as well as mother-to-child transmission of HIV, has the potential for high public health impact.

We chose a rigorous three-arm randomized control trial design to enable comparison between the home-based intervention with the relatively low-cost approaches of HIV self-testing kits and standard of care that do not include extensive health-related counseling. It is important to compare our home-based couple intervention with these approaches for both efficacy and cost-effectiveness since male invitations to come to the clinic are the current standard of care and HIVST has shown promise in boosting male partner and couple testing. This is crucial as policymakers may have a natural reluctance to adopt more complex and expensive interventions without strong evidence that these interventions are more effective than simpler and cheaper alternatives and that they are worth the extra financial investment.

The approaches we are testing to improve HIV couple testing and disclosure to increase the use of PMTCT and family health services are the result of our team’s extensive formative and pilot research [23, 32, 36, 74]. The current intervention innovates in multiple ways. Firstly, this study fills a gap by targeting expectant mothers and fathers as a couple. Recent literature and WHO guidance have called for a renewed emphasis on couples to enhance PMTCT and HIV prevention efforts [75, 76]. A meta-analysis by Crepaz and colleagues found that couple-based interventions were more effective than individual approaches for both HIV testing and nevirapine uptake [77]. However, there are few couple interventions for pregnant women and male partners in low-resource settings [78]. Distinct from existing programs like mothers2mothers [79], our intervention is conducted by a pair of lay health workers (one male and one female) who engage both partners of the couple and foster positive relationship dynamics (e.g., communication) in the promotion of family health.

Secondly, it makes use of a theoretical framework based on the couple interdependence theory [37]. Research has shown that couple relationship factors are associated with health behavior change and health outcomes [80]. Similar associations have been found in HIV research, where partner dynamics influence both prevention and treatment adherence [81]. Yet, couple-based theories are only just beginning to be applied to HIV-related health behavior in sub-Saharan Africa [82, 83]. Home-based HIV counseling and testing have proven to be feasible in Kenya [84–86], and new home-based strategies targeting both pregnant women and partners are showing positive outcomes [87, 88]. The Jamii Bora Study, however, is among the first to rigorously test an intervention based on an interdependence model of communal couple coping and behavior change [37] on PMTCT-related and maternal health outcomes.

Thirdly, this trial attempts to reach beyond the clinic with home-based interventions and with HIV self-testing. Most current couple testing strategies require both partners to come to the clinic, thereby reaching only a minority of couples. Our proposed home-based approach has the potential to reach both pregnant women and male partners in a space that is safe, convenient, inexpensive, and less stigmatizing than men accompanying women to the ANC clinic. Home visits are designed for all pregnant couples (regardless of a woman’s initial HIV test result at the ANC clinic) and include topics important for maternal, paternal, and child health during pregnancy and postpartum. This approach capitalizes on men’s heightened concern for family health during pregnancy [89] and is more likely to engage men than approaches that focus solely on HIV-related health. Additionally, we will compare our home-based intervention to one that relies on the secondary distribution of HIVST by women to their partners, which will expand the evidence base on whether HIVST can play a useful role in PMTCT and HIV risk reduction. This will come at a time when many sub-Saharan African countries are actively developing HIVST policies.

Upon completion of this trial, we will gain evidence of the comparative efficacy of these three different approaches to engaging couples in health behaviors and outcomes, specifically in the context of antenatal care in an area of high HIV prevalence. We will be able to present this evidence to the Kenyan Ministry of Health and partners for potential expansion of effective strategies to sites across the country. Effective strategies can also be adapted for and tested in other similar settings in sub-Saharan Africa, with important potential benefits for maternal, paternal, and infant health.

## Trial status

**Fig. 3:**
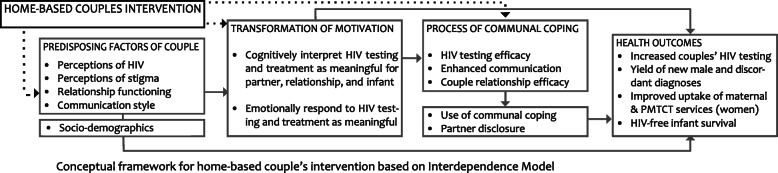
Conceptual framework for home-based couple intervention based on interdependence model

**Fig. 4:**
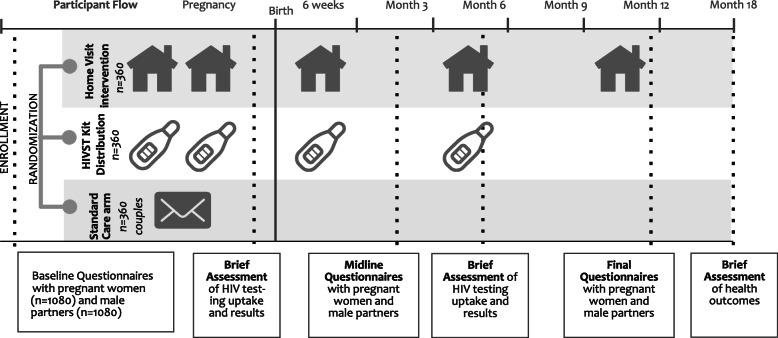
Participant flow

The Jamii Bora Study is currently in month 13 of 60 planned months of recruitment and data collection. The study initiated recruitment on March 20, 2019, and anticipates completion in December 2021. A populated Standard Protocol Items: Recommendations for Interventional Trials (SPIRIT) Checklist and figures and tables for the study intervention designs, conceptual framework, intervention content, and factors and measures are included as additional files (see Figs. 1, 2, 3, and 4 and Tables 1, 2, and 3). The protocol is now at amendment 4, version 6.0 April 04, 2020.

## Supplementary Information

**Additional file 1.** SPIRIT 2013 Checklist.

**Additional file 2.** HIV Test Informed Consent Form.

## Data Availability

Not applicable. No datasets are included in this study protocol.

## Abbreviations

Jamii Bora Study: Testing strategies for couple engagement in PMTCT and family health in Kenya
ART: Antiretroviral therapy
CHTC: Couples’ HIV testing and counseling
HIVST: HIV self-testing kits
PMTCT: Prevention of mother-to-child transmission of HIV
ANC: Antenatal care
MCH: Maternal and child health
Option B+: Lifelong ART for women living with HIV
VL: Viral load
PrEP: Pre-exposure prophylaxis
MoH: Ministry of Health
