# Supplementary material for: Testing strategies for couple engagement in prevention of mother-to-child transmission of HIV and family health in Kenya: study protocol for a randomized controlled trial
Source: Trials. 2021 Jan 6;22:19. doi: 10.1186/s13063-020-04956-1 (PMC7788905; doi:10.1186/s13063-020-04956-1)
Supplement: Supplementary file 2 — Additional file 2. HIV Test Informed Consent Form. [file 13063_2020_4956_MOESM2_ESM.docx]

**HIV TEST INFORMED CONSENT FORM**

**Testing Strategies for Couple Engagement in PMTCT and Family Health in Kenya**

Conducted by the Kenya Medical Research Institute, the University of Alabama at Birmingham

(USA), the University of Michigan (USA), University of Pennsylvania (USA), and University of the Witwatersrand (South Africa).

| **Name** | **Institution** | **Contact** |
| --- | --- | --- |
| Janet M. Turan | University of Alabama at Birmingham (UAB) | 000-1-205-934-6780 |
| Zachary Kwena | Kenya Medical Research Institute (KEMRI) | 0733 333 005 |
| Elizabeth Bukusi | Kenya Medical Research Institute (KEMRI) | 0733 617 503 |
| Lynae Darbes | University of Michigan (UM) | 000-1-734-763-7265 |
| Thomas Braun | University of Michigan (UM) | 000-1-734-936-9844 |
| Abigail Hatcher | University of the Witwatersrand, South Africa | 000-27-84-406-7773 |
| Harsha Thirumurthy | University of Pennsylvania (UPenn) | 000-1-215-898-7136 |
| Maria Pisu | University of Alabama at Birmingham (UAB) | +1 205-975-7366 |

**24-hour Emergency contact number**: 0724445560

***Introduction***

A virus called HIV (Human Immunodeficiency Virus) causes the disease AIDS (Acquired Immunodeficiency Syndrome). Anyone with HIV can spread it to others. It is spread through unsafe sex, sharing needles, or donating blood or other tissues. Infected mothers can spread HIV to their babies. The test for HIV detects the body's reaction to the virus (antibody). It does not detect the virus itself. The decision to be tested for antibody to the virus that causes AIDS is voluntary; you are not required to have the test. This test is being done for a research study.

You should know the advantages and disadvantages of testing before you decide to take the test. Please read this consent form with care so that you can make an informed choice about having the blood test.

***What the test means***

If you test POSITIVE, you have the HIV virus. That means you can pass it to others. The test cannot tell how long a person has been infected. It does not mean that you have AIDS, which is the most advanced stage of HIV infection.

If the test is NEGATIVE you probably do not have the HIV virus. It may mean that you have the virus, but your body has not yet made antibody to fight the virus. It could take up to six months after infection for the test to turn positive. False results are rare. Unclear results are also rare. When a test result does not seem to make sense, we do the test again. We might do another kind of blood test to find out if you are infected or not.

***Procedures***

This is what will happen if you decide to have the test. First, you will meet with a counselor. The counselor will give you more information about the risks and benefits of the test. They will explain the meaning of test results. They will teach you how to reduce the chance of spreading HIV. They will explain the dangers of HIV infection. A finger prick blood will be obtained for the antibody test. We will test your blood for HIV at the study clinic and later in the laboratory in case of invalid results. For participants with invalid HIV results at study clinic, we will make every effort to locate you and give you the definite results. When you learn the test results, you will also be counseled to increase your understanding of HIV transmission and how to reduce your risks of getting or transmitting sexually transmitted diseases by being faithful to one uninfected partner, abstaining from sex if you are diagnosed with a sexually transmitted disease while on treatment, and using a condom consistently and correctly each time you have sex. You will also be counseled about how to notify your sexual partners if your test result is positive

***Benefits of being tested***

The benefits of being tested are very personal. If you are worried about AIDS, you might feel better if you have a negative test. Sometimes knowing that the test is positive can relieve stress. You may want to know your test result before you have sex with a partner. In some cases, test results may help diagnose a medical problem or help you make decisions about your future or on health care. Those who test positive for HIV will be referred to HIV care clinics for further management. There may be other benefits of testing that we don't know about now.

***Risks of being tested***

Learning test results may cause you and your partner severe stress, anxiety and depression. This may result into blaming each other and even cause separation or divorce. Other people

learning about your HIV status may lead to discrimination in travel, work and insurance. You might be tempted to have unsafe sex if the result is negative. This would increase your risk of

getting AIDS. If the results of the test get into the wrong hands, prejudice, discrimination, risk to employment, travel restrictions, and other adverse effects could result. There may be other

risks and stresses of being tested that we don't know about now.

You may get a bruise where the needle enters the vein and there is a small risk of infection. You may feel some pain as the needle enters your vein.

***Information about confidentiality***

Your HIV antibody test results will be held in the strictest confidence, and no identifying information of any kind will be released to any other person or agency without your specific permission in writing. We will not publish or discuss in public anything that could identify you.

Do you have any questions? Do you agree to participate?

Name of researcher Signature Date

***Participant’s Statement***

I have read this form/this form has been read and explained to me. I volunteer to take part in this research. I have had a chance to ask questions. If I have future questions about the research, I can ask one of the investigators listed above. If I have questions about my rights as a research subject, I can contact: The Secretary, KEMRI Ethics Review Committee, P.O. Box 54840-00200, Nairobi; Telephone numbers: 020-2722541, 0722-205901, 0733-400003; Email address: [ERCadmin@kemri.org](mailto:ERCadmin@kemri.org).

Printed name of participant Signature/thump print Date Time

Printed name of witness Signature of witness Date Time

Copies to: Investigator’s files, study participant

**HIV TEST INFORMED CONSENT TO BE A RESEARCH PARTICIPANT (Fomu ya idhini ya kupimwa virusi vya ukimwi)**

Uliofanywa na Taasisi ya Utafiti wa Kimatibabu ya Kenya, Chuo kikuu cha Alabama kule Birmingham (USA),Chuo kikuu cha Michigan (USA), Chuo kikuu cha Pennsylyvania (USA) na Chuo kikuu cha Witwatersrand (South Africa).

| **Name** | **Institution** | **Contact** |
| --- | --- | --- |
| Janet M. Turan | University of Alabama at Birmingham (UAB) | 000-1-205-934-6780 |
| Zachary Kwena | Kenya Medical Research Institute (KEMRI) | 0733 333 005 |
| Elizabeth Bukusi | Kenya Medical Research Institute (KEMRI) | 0733 617 503 |
| Lynae Darbes | University of Michigan (UM) | 000-1-734-763-7265 |
| Thomas Braun | University of Michigan (UM) | 000-1-734-936-9844 |
| Abigail Hatcher | University of the Witwatersrand, South Africa | 000-27-84-406-7773 |
| Harsha Thirumurthy | University of Pennsylvania (UPenn) | 001-1-215-898-7136 |
| Maria Pisu | University of Alabama at Birmingham (UAB) | +1 205-972-7366 |

**24-hour Emergency contact number**: 0724445560

. **Utangulizi**

Virusi ijulikanayo kama HIV (Human Immunodeficiency Virus) husababisha UKIMWI (Ukosefu wa Kinga Mwilini). Mtu yeyote aliye na virusi vya ukimwi anaweza kusambazia wengine. Inaenea kwa njia ya kushiriki ngono bila kutumia kinga, kutumia sindano pamoja na mtu aliyeambukizwa, ama kwa kutoa damu na viungo vingine vya mwili. Mama aliye na virusi anaweza kumsambazia mtoto wake virusi vya ukimwi. Njia ya kupima virusi hutambua mmenyuko wa mwili dhidi ya virusi. Haitambui virusi. Uamuzi wa kupimwa virusi vya Ukimwi inayosababisha UKIMWI ni kwa hiari. Sio lazima upimwe. Kupima virusi vya Ukimwi inafanywa kwa minajili ya utafiti. Ni vizuri ukijua manufaa na mabaya ya kupimwa kabla haujakubali kupimwa. Tafadhali soma nakala ya idhini kwa makini ili ufanye uteuzi mwafaka kuhusu kupimwa damu.

**Maana ya matokeo**

Ukipimwa na upatikane na virusi inamaanisha una Virusi Vya Ukimwi.Hiyo inamaanisha ya kuwa unaweza sambazia wengine. Kipimo hiki hakionyeshi mtu ameambukizwa kwa muda gani. Pia haimaanishi una UKIMWI ambayo ni kiwango cha juu zaidi cha maambukizi ya Virusi Vya Ukimwi.Ukipimwa na hauna virusi, pengine hauna Virusi Vya Ukimwi. Inaweza pia maanisha ya kuwa una virusi lakini mwili wako bado haujaanza kupigana na virusi. Inaweza kuchukua hadi miezi sita baada ya kuambukizwa ili kipimo hiki kionyeshe ya kwamba una virusi. Matokeo yasio ya ukweli ni nadra mno, pia majibu yasioleleweka ni nadra. Iwapo kipimo hakionyeshi matokeo ya kueleweka tutarudi kupima tena. Tunaweza kutumia njia tofauti ya kupima Virusi Vya Ukimwi ili tujue ikiwa umeambukizwa au la.

**Utaratibu**

Yafuatayo yatafanyika ukikubali kupimwa. Kwanza, utakutana na mshauri. Mshauri atakupa habari zaidi kuhusu uzuri na ubaya wa kupimwa. Atakuelezea maana ya matokeo. Atakufunza jinsi ya kupunguza nafasi ya kueneza Virusi Vya Ukimwi. Watakueleza athari za kuambukizwa Virusi Vya Ukimwi. Kidole chako kitadungwa kwa minajili ya kupima. Tutapima ikiwa damu yako ina Virusi Vya Ukimwi katika kliniki ya utafiti na kisha kwa maabara ikiwa tutapata matokeo ambayo hayaeleweki. Tutajaribu vilivyo kutafuta washiriki wa utafiti ambao watakuwa na matokeo isiyoeleweka ili tuwapatie matokeo sahihi. Utakapopata matokeo, utazidi kushauriwa ili uelewe zaidi kuhusu usambazaji na jinsi ya kupunguza nafasi yako ya kupata au kuambukizwa magonjwa ya zinaa kwa kuwa mwaminifu kwa mpenzi mmoja asiyeambukizwa, kuwacha kushiriki kimapenzi iwapo umepatikana na ugonjwa wa zinaa wakati unapata matibabu, na kutumia kondomu/mpira ipasavyo na mfululizo kila mara unaposhiriki ngono. Pia utapata ushauri jinsi ya kueleza washirika wako wa kimapenzi ikiwa matokeo yataonyesha ya kuwa umeambukizwa

**Manufaa ya kupimwa**

Faida ya kupimwa ni binafsi sana. Ikiwa una hofu kuhusu UKIMWI utahisi vyema ukipata matokeo kuonyesha hauna virusi. Wakati mwingine kujua kwamba matokeo inaonyesha una virusi inaweza kurudisha chini hali ya wasiwasi. Unaweza kutaka kujua hali yako kabla ya kushiriki ngono na mpenzi. Wakati mwingine, matokeo inaweza kutatua shida za kiafya ama kufanya uamuzi kuhusu siku zako za usoni ama za huduma za afya. Wale watakaopatwa na virusi vya ukimwi wataelekezwa katika kliniki ya huduma ya HIV ili wapate matibabu. Kuna uwezekano kuwa kuna faida za kupimwa ambazo hatujui kwa sasa.

**Madhara ya kupimwa**

Kujua matokeo inaweza kuwaletea wewe na mpenzi wako hali kali ya dhiki, wasiwasi na huzuni. Hali hii inaweza kuleta kulaumiana na hata kusababisha kuwachana au talaka. Kujulikana kwa hali yako ya virusi na watu wengine inaweza sababisha changamoto kwa usafiri,kuajiriwa na hata kupata bima. Unaweza kudanganyika kushiriki ngono bila kujikinga ikiwa matokeo inaonyesha hauna virusi. Hali hii itaongeza maradufu nafasi zako za kupata ukimwi. Matokeo ikipatikana kwa mikono isiyo halali, chuki, ubaguzi, hatari ya kutoajiriwa, vikwazo kwa kusafiri na matukio mengine mabaya yanaweza kutokea. Kunaweza kuwa na hatari zingine za kupimwa ambazo hatujabainisha kwa wakati huu.Utapata mkwaruzo mdogo unapodungwa sindano kwa mshipa na kuna uwezekano mdogo wa pahali hapo kuambukizwa. Pia utahisi uchungu wakati sindano inaingia kwa mshipa.

**Habari kuhusu siri**

Matokeo yako ya kupimwa UKIMWI itawekwa kwa nji ya siri zaidi na hakuna aina yoyote ya habari za kukutambulisha/kukuhusisha itapewa mtu au kongamano yeyote bila ya sisi kupata ruhusa ya kipekee iliyoandikwa kutoka kwako. Hatutachapisha au zungumzia hadharani kitu chochote kinachoweza kukutambulisha.

Je,una maswali? Je, umekubali kushiriki?

***___________________________ _____________ _______________***

**Jina la Mtafiti Sahihi Tarehe**

***Kauli ya mshiriki***

Nimesoma fomu hii/ Nimesomewa nakala hii na nimeelezwa kwa kina. Nimekubali kushiriki katika utafiti huu. Nimepewa nafasi ya kuuliza maswali. Ikiwa nina maswali baadaye ninaweza kuwasiliana na watafiti waliyo orodheshwa hapo juu. Ikiwa nina maswali kuhusu haki yangu kama mshiriki wa utafiti ninaweza kuwasiliana na karani wa KEMRI Scientific Steering Committee, S.L.P 54840-00200, Nairobi; nambari ya simu 020-272-2541, 0722-205901, 0733-400003; barua pepe: ERCadmin@kemri.org.

| ___________________ | _______________ | _____________ | ____________ |
| --- | --- | --- | --- |
| Chapisha jina la Mshiriki | Sahihi/Alama ya kidole Tarehe | | Saa |
|  |  |  |  |
| Chapisha jina la Shahidi | Sahihi ya Shahidi | Tarehe | Saa |
|  |  |  |  |

Nakala kwa: Faili ya Mpelelezi, Mshiriki wa Utafiti

**HIV TEST INFORMED CONSENT FORM**

**Testing Strategies for Couple Engagement in PMTCT and Family Health in Kenya**

Itimo kod jotim nonro mawuok kar timo nonro mar thieth e piny Kenya, Mbalariany mar Alabama manitiere Birmingham (USA), Mbalariany mar Michigan (USA), Mbalariany mar Pennsylvania (USA), kod Mbalariany mar Witwatersrand (South Africa).

| **Name** | **Institution** | **Contact** |
| --- | --- | --- |
| Janet M. Turan | University of Alabama at Birmingham (UAB) | 000-1-205-934-6780 |
| Zachary Kwena | Kenya Medical Research Institute(KEMRI) | 0733 333 005 |
| Elizabeth Bukusi | Kenya Medical Research Institute(KEMRI) | 0733 617 503 |
| Lynae Darbes | University of Michigan (UM) | 000-1-734-763-7265 |
| Thomas Braun | University of Michigan (UM) | 000-1-734-936-9844 |
| Abigail Hatcher | University of the Witwatersrand,South Africa | 000-27-84-406-7773 |
| Harsha Thirumurthy | University of Pennsylvania (UPenn) | 001-1-215-898-7136 |
| Maria Pisu | University of Alabama at Birmingham (UAB) | +1 205-972-7366 |

**24-hour Emergency contact number**:0724445560

**Weche motelo**

Kute mag ayaki ma iluongo ni HIV (Human Immunodeficiency Virus) kelo tuo mar AYAKI. (Acquired Immunodeficiency Syndrome). Ng’ato ang’ata man gi kute mag ayaki nyalo lande ne jomamoko. Olandore e yor terruok ma ok kare, riwo sindene, kata chiwo remo kod lemo mamoko mag del. Mine man gi kute mag ayaki be nyalo chiwo kutegi ne nyithindgi. Pim kute mag ayaki pimo mana kaka dendi orwako kutegi. Pimni ok fweny kutegi giwegi. Yiero mondo opimi ka in kod kute mag ayaki en kuom hero mari, ok ochuno ni nyaka iyud pimni. Pimni itimo ne wach nonro. Nyaka ing’e ber kod rach mar dhi e pim kapodi ok ing’ado rieko mar dhi e pim. Yie isom oboke ni gi kinda mondo ing’ad rieko mar dhi e pim.

**Gima pim onyiso**

Ka duokoni owuok POSITIVE, in kod kute mag ayaki. Ma nyiso ni inyalo lando kutegi ne ji mamoko. Pimni ok nyal nyiso ni ng’ato osebedo kod kutegi kuom kinde maromo nade. Ma ok onyiso ni in kod ayaki ma en okang’ moseniang’ ka ng’ato osebet kod kutegi.

Ka duokoni owuok NEGATIVE samoro ionge kute mag ayaki. Nyalore be ni in kod kute mag ayaki to dendi podi ok oloso jolweny ma goyore kodgi. Nyalo kawo madirom dweche auchiel bang’ yudo kutegi mondo pim onyis ni kute nitiere. Ka duoko mar pim chal ma ok ochomo yo, wanuoyo pim no kendo. Wanyalo timo kendo kido machielo mar pim mar remo mondo wanon ka igamo kute kata ok igamo.

**Okenge**

Ma e gima biro timore ka iyiero timo pim. Mokuongo, ibiro romo kod jahocho. Jahocho no biro miyi weche ma omedore ewi ber kod rach pim. Gibiro leroni tiend duoko mar pim. Gibiro puonji kaka inyalo duoko chien thuolo mar lando kute mag AYAKI. Gibiro leroni rach mar gamo kute mag ayaki. Ibiro golo remo ewi koki ne pim mar kute mag ayaki. Wabiro pimo rembi ne kute mag AYAKI e klinik mar nonro kendo bang’e e labaratori kapo ni duoko ok owuok manenore maler. Ne jochiwre ma duokogi ok nenre maler e klinik mar nonro, wabiro timo duto mondo wamanyi gi duoko ma oriere. Ka ipuonjori duoko mar pim, ibiro bende miyi hocho mondo omed ng’eyoni kaka kute mag AYAKI landore to kod kaka inyalo duoko chien thuolo mar yudo kata chiwo tuoche mag nyaye kuom bedo ratiro kod jaherani achiel maonge kutego, weyo chuth bedo e achiel e ringruok e kinde ma in e thieth ka oyudi ni intie kod tuoche mag nyaye kod tiyo kod rabo yunga kinde duto kendo e yo ma owinjore samoro amora ma ibedo e ringruok achiel. Ibiro bende miyi hocho kuom kaka inyalo yango ne joherani duoko mar pim ka owuok ni kute mag ayaki nitiere.

**Ber mar pim**

Ber mag pim en gima ng’ato winjo owuon. Ka wach mag kute mag ayaki osebedo ka thagi to inyalo winjo maber ka iyudo ni ionge kod kute mag ayaki. Seche moko bende ng’eyo ni in kod kute mag ayaki nyalo miyi kwe, inyalo dwaro ng’eyo chalni mar kute mag ayaki kapodi ok ibedo e achiel gi osiepi/jaodi. Seche moko duoko mar pim mar kute mag ayaki nyalo konyo fuenyo tuoche mamoko kata nyalo konyi e ng’ado rieko ewi ngimani mar thieth e kinde mabiro. Jogo maduokogi owuok ni gin kod kute mag ayaki ibiro mi oboke mar ote kar thieth ne kony ma omedore, bende nyalo bedo ni nitiere ber moko mag pim ma ok wang’eyo sani.

**Chandruok manyalo wuok e pimruok**

Ng’eyo duoko mar pim nyalo miyi in kod jaodi paro mang’eny, dang’ni to gi achiedh nadi. Ma gikone nyalo kelo seyo nyawadu kendo nyalo kelo pogruok kata weruok. Jok moko be ka ong’eyo chalni mar kute mag ayaki to nyalo kwedi ka gi dhi e wuoth kata e tich kata “bima”. Gimoro nyalo temi mondo iriwri gi ng’ato ka iyudo ni ionge kod kute mag ayaki to ma biro medo thuolo mari mar yudo kute mag ayaki. Kaponi dwoko olwar e lwet ji mamoko ma ok owinjore, sunga, akwede, hinyruok korka tich, akwede mag wuoth kod rach ma omedore nyalo bedoe. Nyalo bet ni nitie chandruok moko ma nyalo wuok bang’ duokoni mar pim mar kute mag ayaki ma podi ok wang’eyo sani. Inyalo yudo hinyruok kama sindan donjo godo e ler kendo nitie rach matin mar yudo tuo. Inyalo winjo rem matin seche ma sindan donjo e ler.

**Weche ewi maling’ling’**

Pim ni mar kute mag ayaki ibiro tim maling’ling’ kendo onge wach moro amora ma inyalo fuenyigo ma ibiro miyo ni ng’ato machielo ka ok ichiwo yie mari eyor ndiko barua. Ok wabindiko kata lando e lela gima nyalo miyo ng’ato fuenyi.

Be in gi penjo moro amora? Iyie mar bedo e nonroni?

___________________ _________________ ____________

Nying jatim nonro Seyi Tarik

**Wach mar jachiwre**

Ase somo obokeni/obokeni ose somna ma olerna maber. Achiwora mar bedo e nonroni, osemiya thuolo mar penjo, ka an gi penjo moko bang’e kuom nonroni to abiro penjo achiel kuom jotim nonro ma nying gi ni malo kanyo. To ka an gi penjo kuom ratiro maga kaka jachiwre e nonroni, to anyalo tudora gi jagoro mar KEMRI Ethical Review Committee P.O. Box 54840-00200, Nairobi; Namba simu 020-2722541, 0722205901, kata 0733400003; Email address: [ERCadmin@kemri.org](mailto:ERCadmin@kemri.org)

| ___________________ | __________________ |  | |
| --- | --- | --- | --- |
| Nying jachiwre | Seyi/lwet jachiwre | Tarik | Saa |
| __________________ |  | ____________ ________ | |
| Nying janeno | Seyi/lwet janeno | Tarik | Saa |

Oor oboke ni: efaend jatim nonro, jachiwre e nonro.
